# Anti-NMDA Receptor Encephalitis in the Polar Bear (*Ursus maritimus*) Knut

**DOI:** 10.1038/srep12805

**Published:** 2015-08-27

**Authors:** H. Prüss, J. Leubner, N. K. Wenke, G. Á. Czirják, C. A. Szentiks, A. D. Greenwood

**Affiliations:** 1 German Center for Neurodegenerative Diseases (DZNE) Berlin, Germany; 2 Department of Neurology, Charité – Universitätsmedizin Berlin, Berlin, Germany; 3 Department of Wildlife Diseases, Leibniz Institute for Zoo and Wildlife Research, Berlin, Germany

## Abstract

Knut the polar bear of the Berlin Zoological Garden drowned in 2011 following seizures and was diagnosed as having suffered encephalitis of unknown etiology after exhaustive pathogen screening. Using the diagnostic criteria applied to human patients, we demonstrate that Knut’s encephalitis is almost identical to anti-NMDA receptor encephalitis which is a severe autoimmune disease representing the most common non-infectious encephalitis in humans. High concentrations of antibodies specific against the NR1 subunit of the NMDA receptor were detected in Knut’s cerebrospinal fluid. Histological examination demonstrated very similar patterns of plasma cell infiltration and minimal neuronal loss in affected brain areas. We conclude that Knut suffered anti-NMDA receptor encephalitis making his the first reported non-human case of this treatable disease. The results suggest that anti-NMDA receptor encephalitis may be a disease of broad relevance to mammals that until now has remained undiagnosed.

Rarely has an animal gained as much worldwide attention during its lifetime as the polar bear (*Ursus maritimus*) Knut, born 2006 in the Berlin Zoological Garden. Knut’s popularity resulted in the most profitable period in the Zoo’s 163-year history. In 2011 in front of running cameras, Knut drowned after he suffered epileptic seizures and fell into the enclosure’s pool. Pathological analyses revealed that Knut suffered encephalitis which caused the seizures^1^. An exceptionally extensive investigation for infectious causes for a single animal failed to demonstrate a bacterial, viral or parasitic cause for the encephalitis. Highly detailed pathological investigations, histology, extensive serology, thirty five pan-PCR assays, two pathogen microarray systems, and shotgun sequencing on both the GSF FLX and Illumina HiSeq systems only revealed a serological response to influenza A and a novel endogenous retroviral group, neither of which would have been responsible for Knut’s symptoms^1^,^2^. Thus, a diagnosis of “encephalitis of unknown etiology” was established.

In human patients it has become clear since 2010 that the vast majority of patients with encephalitis of unknown etiology, after exclusion of infectious causes, results from an autoimmune disease called anti-NMDA receptor (NMDAR) encephalitis^3^,^4^,^5^. It is a severe disease associated with highly specific autoantibodies against the NR1 subunit of the NMDA type glutamate receptor^6^. It follows a specific multistep clinical pattern starting with prodromal signs (such as headache, nausea, low-grade fever) and psychosis (hallucinations, delusions, suicidal thoughts) progressing to epileptic seizures, reduced levels of consciousness, dyskinesias, autonomic dysfunction and hypoventilation. Within a few years since its discovery, more than 1000 patients have been described^7^, making it a more common disease than other encephalitides, such as herpes simplex virus encephalitis. The discovery has revolutionized the field of modern clinical neurology. However, while another form of anti-receptor autoimmunity with LGI1 antibodies has been observed recently in domestic cats^8^,^9^, the more common anti-NMDAR encephalitis has only been considered a human disease leaving open the possibility that an entire field of encephalitis relevant research has gone unexplored in domestic and wild animals.

Prior to the identification of NMDAR antibodies, human patients with this characteristic disease were often diagnosed with encephalitis of unknown etiology and thus, we tested the hypothesis that Knut’s encephalitis may have been caused by NMDAR antibodies which would make his the first diagnosed non-human case and suggest that anti-NMDAR encephalitis is a general disease of mammals.

## Results

### Autopsy demonstrated encephalitis

**Figure 1:**
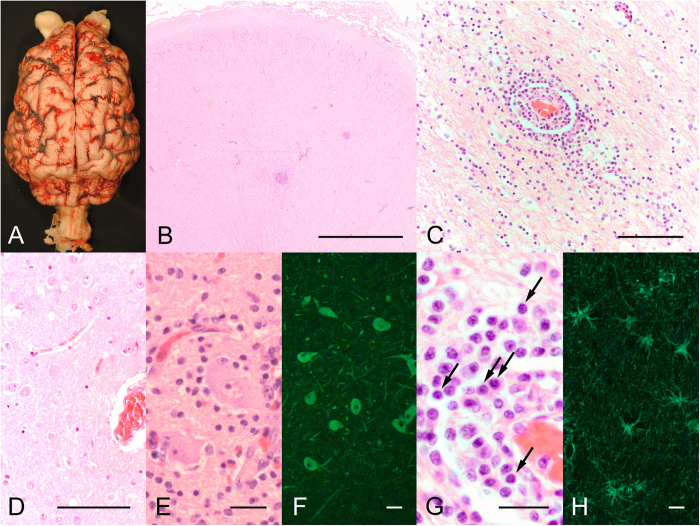
Brain examination demonstrates encephalitis with inflammatory infiltrates. Macroscopic view of Knut’s brain (**A**). Survey micrograph of the brainstem showing areas of “normal appearing” brain and areas with infiltrating immune cells (**B**). Higher magnification of a cerebral brain vessel in the hippocampal formation demonstrating infiltration of immune cells into the brain parenchyma (**C**). In contrast, a brain section of a control male polar bear shows absence of immune cells in the tissue (**D**). Despite widespread inflammatory infiltrates, neurons show mainly intact size and morphology (**E**: H&E staining, **F**: MAP2 immunofluorescence). Inflammatory infiltrates contain a high number of plasma cells (**G**, arrows). GFAP immunostaining demonstrates reactive gliosis in areas of inflammation (**H**). Bars represent 1000 μm in **B**, 100 μm in **C,D**, and 25 μm in **E–H**.

The brain was removed (Fig. 1A) and analyzed by histology. H&E stained sections of brainstem and cerebrum demonstrate a patchy distribution of infiltrating immune cells among normal brain tissue (Fig. 1B). In some areas, pronounced inflammation was detected with inflammatory infiltrates transmigrating from small brain vessels into the parenchyma, although the density of immune cells was lower than observed in infectious encephalitis (Fig. 1C). The cells were not clustered in the vessel wall as in vasculitis. In a normal polar bear, the brain parenchyma lacks immune cells, as shown on sections of a male polar bear that died after foreign body ingestion (Fig. 1D). In healthy and inflamed brain areas, the neurons were largely intact, arguing for a disease mechanism that did not primarily depend on cytotoxic T-cells (Fig. 1E,F). Instead, numerous plasma cells were detected around vessels and within the parenchymal infiltrates (Fig. 1G, arrows). Although the plasma cells do not always have the appearance expected for human patients, they are nearly identical to plasma cells observed in other carnivores such as dogs^10^. Unspecific reactive gliosis was demonstrated with immunostaining of GFAP (Fig. 1H). Apart from the brain, no obvious abnormalities were detected in other organs and no tumors were detected at necropsy.

### Polar bear NMDA receptor homology

**Figure 2:**
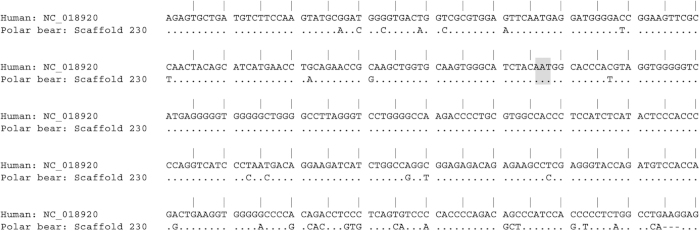
NMDAR sequence homology between human and polar bear. Alignment of the extracellular N-terminal part of the human NR1 subunit of the NMDA receptor with the polar bear genome scaffold 203 demonstrating high sequence conservation across species. Identical sequences are marked by a dot. Different bases between human and polar bear are given, deletions are shown as a dash. The sequence highlighted in grey represents the epitope recognized by the majority of human NMDAR autoantibodies.

Sequence analysis of the human NR1 subunit of the NMDA receptor showed very high homology with portions of the polar bear genome scaffold 203^11^, in particular in the amino terminal extracellular region of the protein containing amino acid N368, which is known to represent the major epitope in anti-NMDAR encephalitis^12^ (Fig. 2).

### Knut’s CSF contains very high levels of NMDAR autoantibodies

**Figure 3:**
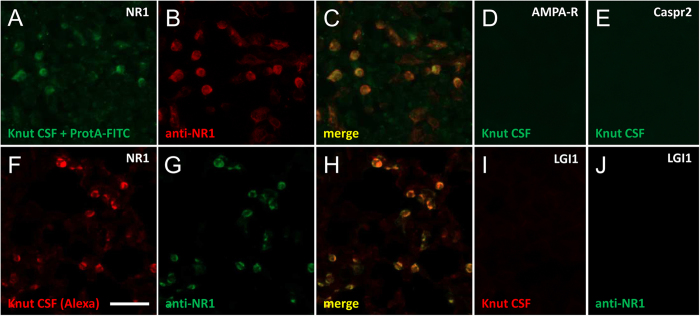
Immunoglobulins from Knut’s CSF specifically bind to NMDAR-transfected HEK cells. Protein A/FITC-labeled polar bear CSF immunoglobulins specifically bind to NMDAR-transfected HEK cells (**A**), similar to a commercial anti-NR1 antibody (**B)**, merged image in (**C**). In contrast, no binding of Knut’s CSF was observed on similarly transfected HEK cells expressing AMPA receptors (**D**), Caspr2 (**E**) or further proteins (not shown). Using an alternative strategy, polar bear CSF was directly labelled with Alexa-594 dyes and probed on transfected cells. Again, NMDAR-transfected cells were specifically labeled (**F**); commercial control antibody in (**G**), merged image in (**H**), while no staining was observed on LGI1-transfected cells (**I**); anti-NR1 antibody in (**J**) and further controls (not shown). Bar in F represents 100 μm for (**A–J**).

The gold standard for testing patients for the presence of NMDAR autoantibodies is the combined use of tissue immunohistochemistry and cell-based assays in which the protein of interest is transiently expressed in HEK cells^6^,^13^. Therefore HEK cells were transfected with GRIN1 cloned into the pBudCE4.1 vector. Cells expressed high levels of NMDAR protein, in contrast with control cells transfected with the vector only. Due to the lack of anti-polar bear secondary antibodies, we used two independent strategies to label CSF immunoglobulins. First, Knut’s CSF was incubated with FITC-tagged protein A. Second, the antibodies were covalently bound to Alexa-594. Both, the protein A-based assay (Fig. 3A–C) and the Alexa coupling (Fig. 3F–H) showed identical results with strong binding to HEK cells expressing the NMDAR, were double-positive with commercial anti-NR1 antibodies, and absent on untransfected cells. Antibody titers were very high, i.e. a positive signal was still detectable when diluting the initial CSF concentration by more than 1:1,000, thus exceeding the CSF antibody titers seen in many patients with anti-NMDAR encephalitis.

Both stainings were verified on Euroimmun BioChips with strong signal on NR1-transfected cells, but no signal on HEK cells transfected with AMPA receptors (Fig. 3D), Caspr2 (Fig. 3E), LGI1 (Fig. 3I), or GABAb receptor (not shown) which are all targets in distinct forms of human autoimmune encephalitis^14^. Thus, with five unrelated receptors tested for reactivity, only NMDAR produced a response in Knut.

### Antibody binding to brain sections

**Figure 4:**
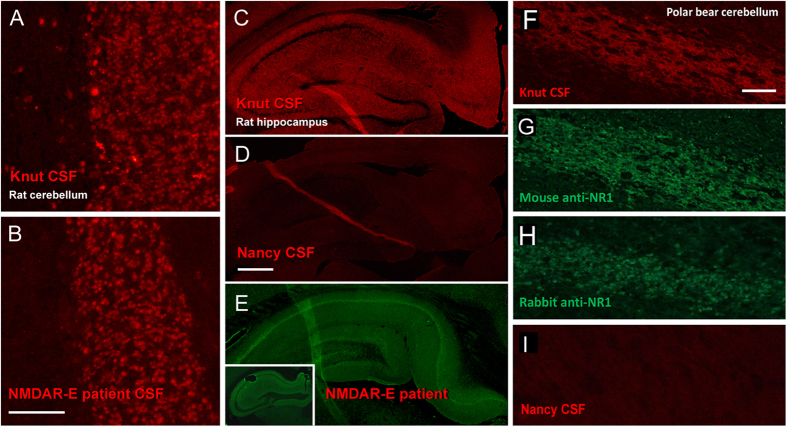
Knut’s antibodies demonstrate the characteristic labeling of neurons known from NMDAR encephalitis. Knut’s antibodies strongly bind to the granule cell layer of rat cerebellum (**A**), a pattern that is characteristic for the staining of CSF from human patients with anti-NMDAR encephalitis (**B**). Also, the typical neuropil staining can be found on hippocampus sections (**C**), while it is absent when testing CSF from a control polar bear without encephalitis (**D**). The immunohistochemistry pattern of Knut’s CSF is very similar to patient CSF staining on rat brain (**E**) or mouse brain sections (**E**, insert). Alexa-594-coupled CSF antibodies further show specific immunostaining on cerebellar sections of Knut’s brain (**F**), which is identical to the pattern when using commercial mouse (**G**) or rabbit anti-NMDAR antibodies (**H**) on polar bear brain, but not with control polar bear CSF (**I**). Bar in A represents 100 μm (**A,B**), in D 500 μm (**C–E**), in F 200 μm (**F–I**).

Antibodies from patients with anti-NMDAR encephalitis are known to strongly bind to rat brain sections, particularly in cortical, hippocampal and cerebellar areas^6^. Knut’s antibodies exhibited an identical labeling pattern as human patients (Fig. 4B) showing intense labeling of rat hippocampus and cerebellum, such as in the cerebellar granule cell layer (Fig. 4A) or hippocampal neuropil (Fig. 4C). CSF from the male polar bear (which died of foreign body ingestion) for which histology was compared to Knut was not available. However, CSF from a second polar bear, Nancy, which died of gastric torsion, did not react at all with rat hippocampus (Fig. 4D) and cerebellum demonstrating reactivity was unique to Knut. As observed in human patients, NMDAR antibodies stain broadly in the hippocampus. Knut’s pattern was very similar to previously reported anti-NMDAR encephalitis cases^6^ or to experimental staining performed in the current study (Fig. 4E, insert shows staining on mouse brain). However, we did observe a difference in staining in the hilum of hippocampus which exhibited reduced staining with Knut’s CSF but staining nonetheless. Human CSF produced less staining in our experiments and has been reported not to bind to this brain region in humans^15^. One possible explanation is that in addition to NMDAR antibodies, another anti-receptor antibody is present in Knut’s CSF. However, if this is the case, it would represent a minor component as NMDAR antibody concentrations were very high, staining was reduced in hilum and it would not represent one of the common human encephalitis antibodies as we excluded antibodies against AMPA receptors, Caspr2, LGI1, and GABAb receptor (Fig. 3). Another likely explanation for the observed pattern is species-specific differences in NMDAR binding by polar bears antibodies. Further molecular immunological studies on polar bears will be needed to clarify this point. We further tested whether Knut’s antibodies bind to his own cerebellum. Indeed, strong staining was detectable in the large fiber tracts of the cerebellum with an axonal distribution (Fig. 4F). As the binding pattern is slightly different to rat brain, we confirmed the specificity by using a monoclonal mouse anti-NR1 (Fig. 4G) and a polyclonal rabbit anti-NR1 antibody (Fig. 4H) which gave identical results on the polar bear brain section. CSF from polar bear Nancy showed no reaction with the tissues (Fig. 4I).

## Discussion

Prior to this study, the death of Knut represented an unsolved mystery given the lack of any pathogen detected subsequent to intensive investigation^1^. Generally, the diagnosis of anti-NMDAR encephalitis in human patients is based on those who have (i) clinical signs of encephalitis (epileptic seizures, reduced levels of consciousness, cognitive or mood changes), (ii) evidence of brain inflammation (MRI abnormalities, CSF inflammation, or positive biopsy/autopsy), (iii) exclusion of viral/bacterial causes, and (iv) detection of CSF (+/− serum) antibodies against the NMDA receptor. Thus, the presence of seizures, inflammatory infiltrates in the brain, exclusion of an infectious etiology, and very high levels of NMDAR antibodies allow a firm diagnosis of anti-NMDAR encephalitis in Knut. The lack of symptoms and immunostaining from CSF from the control female, a polar bear which died of gastric torsion and would not be expected to have autoimmune disease, and lack of histological evidence for autoimmune disease in an unrelated male polar bear, which died after foreign body ingestion, further strengthens the argument that Knut alone exhibited characteristics of anti-NMDAR encephalitis. In addition to the antibodies, the cellular composition of inflammatory infiltrates resembles the pattern known from human patients with anti-NMDAR encephalitis, characterized by only very limited neuronal pathology and a low density of inflammatory cells^16^. Although long term observation of Knut’s epileptic syndrome was not possible, video footage taken at the time demonstrated focal seizure initiation and generalization to grand mal seizures, consistent with the epileptic syndrome in human patients. The presence of antibodies against influenza A virus in the polar bear is also a known finding in patients with anti-NMDAR encephalitis^1^,^4^,^17^.

The direct pathogenic role of NMDAR antibodies has been verified in numerous studies demonstrating immunoglobulin-induced internalization of surface NMDARs^18^. Recently it has been demonstrated that laboratory mice infused with CSF from anti-NMDAR encephalitis patients exhibit altered memory and behavior in addition to histopathological features of human disease demonstrating a direct role for antibody mediated loss of NMDAR and disease^19^. As Knut’s antibodies specifically bind to NMDARs and the immunostaining pattern on brain sections is nearly identical to what is seen with CSF of human anti-NMDAR encephalitis patients, we propose that the antibodies also caused the encephalitis that resulted in the polar bear’s death although direct confirmation is not possible. Autoimmune encephalitides, in particular anti-NMDAR encephalitis, respond well if immunosuppressive therapy is started early and sufficiently aggressive. It is tempting to speculate that anti-NMDAR encephalitis in polar bears is similarly treatable and that had he not drowned, Knut could have survived the condition, in particular as administration of high-dose corticosteroids can be done routinely in captive animals.

Although encephalitis is common among domestic and captive wild animals, the causative agent is often undiagnosed and thus the cases remain unreported or the pathology is atypical such as lack of inclusion bodies in polar bears and rhinoceros infected with zebra equine herpesviruses^20^. Even in cases where infectious causes are suspected, additional autoimmune mechanisms might participate in disease progression, similarly to what has been shown for the brain infection with herpes viruses and subsequent anti-NMDAR encephalitis^21^. If confirmed, this would guide new approaches to managing encephalitis in wild and domestic animals, in particular in species with high conservation value, such as polar bears and rhinoceros.

Based on the results presented here, one could anticipate that in addition to anti-NMDAR encephalitis, other forms of autoimmune encephalitis known from humans will be discovered in wild mammals and will have to be considered in the field of zoo and wildlife medicine in the future^14^. Knut’s anti-NMDAR encephalitis suggests that antibody-mediated autoimmunity might be a far more common disease in mammals than previously thought.

## Methods

### Ethics Statement

The experiments undertaken in this project were approved by the Internal Ethics Committee of the Leibniz Institute for Zoo and Wildlife Research (IZW), Approval No. 2012-04-01. All experiments undertaken were in accordance with the approved guidelines. All experimental protocols were performed with the approval of the Leibniz Institute for Zoo and Wildlife Research and the Charité University Medicine Berlin.

### Protein A-FITC labeling of antibodies

In contrast to protein-G, staphylococcal protein A (SP-A) is known to bind polar bear IgG^22^. 100 μl of cerebrospinal fluid (CSF) and 50 μg FITC-conjugated SP-A (Sigma, #P5145) were incubated for 2 h in the dark at room temperature and diluted to 500 μl with PBS. The mixture was re-concentrated and unbound FITC–SP-A removed using 100 kDa Amicon filter units (Millipore).

### Alexa-594 labeling of antibodies

After establishing optimal labeling conditions with CSF of patients with anti-NMDAR encephalitis, polar bear CSF was conjugated with N-Hydroxysuccinimid-ester of Alexa Fluor 594 (Life Technologies). For this step, 110 μg of Alexa-594 and 100 μl polar bear CSF were incubated for 1 h at room temperature on a shaker at 200 rpm. The sample was diluted in PBS to 500 μl and purified using Amicon columns as above.

### Cell transfection and antibody detection

The cDNA of GRIN1 was kindly provided by Prof. Dr. Wanker (MDC, Berlin) and cloned into pBudCE4.1 (Life Technologies). NR1 DNA (1 μg) was mixed with 3 μg PEI and 100 μl 150 mM NaCl, vortexed and incubated for 10 min, and HEK293 cells were transiently transfected. Two days later, HEK cells on cover slips were fixed with methanol at −20 °C for 4 min. In addition, HEK cells transfected with a different NMDAR clone, leucine-rich glioma-inactivated 1 (LGI1), contactin-associated protein 2 (Caspr2), α-amino-3-hydroxy-5-methyl-4-isoxazolepropionic acid (AMPA) receptor, and gamma-aminobutyric acid b (GABAb) receptor were used (Autoimmune-Enzephalitis-Mosaik 1, Euroimmun, Lübeck, Germany). For staining with polar bear CSF, cells were washed in PBS, preincubated with 5% normal goat serum containing 2% bovine serum albumin and 0.1% Triton X-100, and incubated with CSF starting at a 1:5 dilution overnight at 4 °C. Sections were washed in PBS and coverslips mounted with Immu-Mount (ThermoScientific). No polar bear serum was available for antibody detection as blood from Knut was coagulated and autolysed as described^1^. Double-labeling of transfected cells was performed using commercial antibodies: monoclonal mouse anti-NR1 (1:100, Synaptic Systems).

### Immunohistochemistry

Paraffin-embedded bear brain sections were deparaffinated with xylene and rehydrated. Antigen retrieval was performed by boiling in 0.1 M citrate buffer pH 6 for 10 min. Rat brain sections were used after PFA fixation. Tissue was permeabilized in 0.1% Triton X-100 in PBS for 20 min and blocked in 10% normal goat serum for 30 min. Polar bear or human CSF was diluted 1:10 and sections incubated overnight at 4 °C. Further stainings included rabbit polyclonal anti-NR1 (1:100, Synaptic Systems), anti-GFAP (1:2,000; NeuroMab) anti-MAP2 (1:500, Millipore).

### Sequence analysis

Using the human NMDA receptor mRNA sequences as a query, the polar bear genome^11^ was examined for genomic scaffolds containing the polar bear NMDAR ortholog. Scaffold 203 of the current polar bear genome contained a sequence with strong (>90%) identity with the human NMDA receptor. The polar bear sequence was used as a blastn query and the human genome scaffold sequence was aligned to the polar bear sequence using BioEdit version 7.0.9.

## Additional Information

**How to cite this article**: Prüss, H. *et al*. Anti-NMDA Receptor Encephalitis in the Polar Bear (*Ursus maritimus*) Knut. *Sci. Rep.* **5**, 12805; doi: 10.1038/srep12805 (2015).
